# Diabetes related health knowledge, attitude and practice among diabetic patients in Nepal

**DOI:** 10.1186/s12902-015-0021-6

**Published:** 2015-06-05

**Authors:** Anju Gautam, Dharma Nand Bhatta, Umesh Raj Aryal

**Affiliations:** Department of Public Health, Pokhara University, Nobel College, Sinamangal, Kathmandu, Nepal; Faculty of Medicine, Epidemiology Unit, Prince of Songkla University, Songkhla, Thailand; Department of Community Medicine, Kathmandu Medical College, Kathmandu, Nepal

**Keywords:** Diabetes, Knowledge, Attitude, Practice, Nepal

## Abstract

**Background:**

Globally, diabetes is the top priority chronic disease. Health literary would be cost effective for prevention and control of diabetes and its consequences. This study was conducted to determine the level of diabetes related health knowledge, attitude and practice (KAP) among diabetic patient and factors associated with KAP.

**Methods:**

An institutional based cross-sectional study was conducted using a non-probability sampling technique to select the diabetic patients. A total of 244 diabetic patients were interviewed from July to November 2014. Data was collected by face to face interview using structured interviewer rater questionnaires. Relative risk ratio (RRR) and 95 % confidence interval (CI) of associated factors were estimated by a stepwise likelihood ratio method with multinomial logistic regression.

**Results:**

More than half (52.5 %) of all patients were female, 18 % were illiterate, and 24.6 % were from rural residence. The diabetes related risk factors were common among diabetic patients; 9.8 % smoker, 16 % alcohol drinking, and 17.6 % reported low or no physical activity. Median score for knowledge, attitude, and practice were 81, 40 and 14 respectively. Among all patients, 12.3 %, 12.7 % and 16 % had highly satisfactory knowledge, attitude and practice respectively. Using highly insufficient knowledge as the baseline, the likelihood of having a level of highly sufficient knowledge was 17 times higher among patients who have graduated and above level of education compared to those who were illiterate. Albeit this value was comparatively lower than insufficient level of knowledge. The probability of having a sufficient level of practice among diabetic patient with a history of smoking was 0.10 times lower than in patient with no history of smoking.

**Conclusions:**

Our study reveals a variation between diabetes related health knowledge, attitude and practice in Nepal among those who are affected by diabetes. Our results show the potential diabetes health literacy needs to be improved or developed for better health promotion.

## Background

Globally, diabetes has been established as a prototypical chronic disease that has affected 347 million people in 2008 [1] and 387 million in 2014 [2]. Among them 77 % of the diabetic people reside in low and middle income countries (LMICs) and 8.3 % was adult population [2]. Up to 2035, 592 million peoples will suffered from diabetes and among them 11 % will be adults [2]. Prevalence of diabetes in South-East Asia is 8.33 % and the national prevalence of Nepal is 4.58 % [2]. In 2014 diabetes was the cause of 4.9 million deaths and was accountable for 11 % of the total global health expenses [2]. More than 80 % of diabetes deaths occur in low- and middle-income countries [3]. By 2030, diabetes will be the 7^th^ leading cause of death [4].

Diabetes can play the vital role in the cause of morbidity and mortality through continued clinical consequence and mortality from the effect on kidney, cardiac functions, renal failure, visual impairment and blindness [5–9]. Evidence from the existing findings shows the increasing trend of global diabetes epidemic need to raise alarm with its risky effects on health cost, health care resources and national health budgets, quality of life, life expectancy and overweight [10–13]. Finding reveals the risk of tuberculosis is three times higher in diabetic patients [14]. Modifiable risk factors are associated with morbidity and mortality of the non-communicable diseases (NCDs) including diabetes. Most of the risk factors blood pressure, tobacco use (9 %), alcohol use, physical inactivity (6 %), unhealthy diet, overweight, and obesity (5 %) are accountable for NCDs related deaths and disabilities [15]. Approximately 20 min daily moderate physical activity can reduce 27 % risk of diabetes and help to reduce weight [15, 16].

Known modifiable risk factors can be reduced and controlled by patients themselves through effective education and enhanced knowledge. Despite the known prevalence of diabetes, there are numerous number of undiagnosed and live births diabetes which incline them to succeeding to diabetes [2]. This figure indicated that the diabetes patient should have good knowledge, attitude and practices. Previous literatures suggested that the low level of health literacy is associated with adverse effect on care, health outcomes and is also social determinant in low-and-middle-income countries [17–20]. Effective management of disease, control of risk factors, diagnosis and prevention awareness are associated with knowledge, attitude and practice of diabetic patients [17–19, 21]. Previous study finding reveal that person who have good knowledge and education have good care of diabetes [18].

KAP related to diabetes could be the helpful for early case detection, prevention and minimize the consequences. KAP related studies reveals that the very poor or low level of knowledge, attitude and practice among diabetes patients [21, 22]. There is sparse literature on KAP about diabetes among diagnosed diabetic patients in Nepal, such type of studies are significant for the use of control and prevention of disease consequences strategies in resource poor countries. Therefore, the objective of our study was to determine KAP among diabetic patients and assess the association of KAP.

## Methods

### Study design, settings and participants

An institutional based, cross-sectional study was conducted in Diabetes, Thyroid and Endocrinology center at Kathmandu metropolitan city of Nepal among diabetic patients from July to November 2014. American diabetic association’s criteria were followed for diagnosis and classification of diabetes [23]. Kathmandu is the international hub and capital city of Nepal which has the largest population density [24]. The study was conducted among 244 diabetic patients, selected by using a consecutive sampling technique. All the patients were considered for inclusion if they were diagnosed any types of diabetes by Endocrinologist declared with blood examination, had received treatment for more than six months, and who had given informed consent. Furthermore, patients were only included if they were not receiving any other treatment or therapy, and had no major psychiatric problem.

We determined that 250 diabetic patients would be satisfactory sample size after adding 5 % non response. It would be sufficient to decide association with other variables with 95 % confidence interval and 90 % power and were assumed that 40 % have poor knowledge and 20 % have poor attitude. Non response rate was 2.4 % and finally we had 244 patients agreed to participate.

### Interviews

Two public health undergraduates carried out the interviews. Structured interviewer rater questionnaires were used for data collection. The interviews were conducted in separated room at the Diabetes, Thyroid and Endocrinology center where patients came for follow up checkup. All the tools were firstly constructed in English, and then translated in Nepali, and again retranslated into English by experts. Questionnaires were pre-tested with diabetic patients in a private hospital at Kathmandu and the required ambiguous alters in the questionnaire was corrected.

### Outcome measures

Knowledge, attitude and practice (KAP) are the primary outcome variables in this study. The KAP questionnaire was prepared from “Garcia and associates for the diabetes self-management project at Gateway community health center’s (Patients’ diabetes knowledge questionnaire)” [25] and university of Michigan diabetes research and training center’s “diabetes attitude survey” [26] after necessary modifications in Nepalese context.

Knowledge was measured using a 30-item questionnaire relating to general knowledge of diabetes (diet patterns, medications, investigations, exercises); danger signs (older age, obesity, genetics, pregnancy etc.); symptoms and complications (problems in kidney, eyes, appetite, physiological changes etc.); treatment and management (life style, drinking, smoking, personal hygiene etc.) with 3-point Likert type scale ranging from 1 (do not know as one) to 3 (correctly know). Scores ranges from 30 to 90 and higher score indicating higher level of knowledge. Example of the asked questions included “Do you think that diabetes affect the blood circulation?”; “Do you think that obesity is the risk for diabetes?” and “Do you think that diabetes affect the eyes?” Internal consistency of the tools for knowledge was measured by Cronbach’s alpha = 0.67 (95 % Confidence Interval: 0.61 to 0.73).

Attitude was measured using a 10-item questionnaire with 5-point Likert type scale ranging from 1 (strongly disagree) to 5 (strongly agree). Scores ranges from 10 to 50 and higher score indicating higher level of attitude. Example of the asked questions included” In general I believe that, “Controlled diet and regular exercise helps in maintenance of blood glucose.” and “Diabetic patient are more responsible than the doctor and family in the care of diabetes.” Cronbach’s alpha was 0.49 (95 % CI: 0.38 to 0.60) for attitude tools for internal consistency.

Practice was measured using a 6-item questionnaire with mixed type of response ranging from 1 (less or no practice) to 5 (more or always practice). Scores ranges from 6 to 23 and higher score indicating higher level of practice. Example of the asked questions included “do you exercise?” (Coded as yes =2 and no or some time = 1); “How often do you eat fruits in your meal?” (Coded as daily = 3, weekly once = 2, sometime or not sure = 1); “How often do you eat green vegetables in your meal?” (Coded as daily = 3, weekly once = 2, sometime or not sure = 1); “How often do you check your blood pressure?” (Coded as monthly = 4, once in a three month = 3, once in a six month = 2, yearly = 1, never =0). Eye examination and other laboratory examinations were coded as the similar fashion of blood pressure variable. Cronbach’s alpha was 0.44 (0.31 to 0.57) for practice tools for internal consistency.

The total score of KAP was classified into next five categories based on the quintile scores and coded as ≤ 20 % “highly insufficient”, 21- 40 % “insufficient”, 41- 60 % “sufficient”, 61- 80 % “satisfactory” and >80 % “highly satisfactory” [27, 28].

### Explanatory variables

Socio-demographic information (age, sex, ethnicity, religion, occupation, residence, education), family history of diabetes, type of diabetes, history of alcohol, history of smoking, and physical activities are considered as explanatory variables for knowledge, attitude, and practice/behavior. Education level was coded as cannot read and write (illiterate), literate (can read and write but received no formal education), primary level (received education up to year five) and secondary or higher (received education more than or equal to class six). Family history of diabetes was measured using a question; “have your any family member (mother or father,) had diabetes?” History of smoking, alcohol, and physical activity has been measured as during life time. In addition, knowledge, attitude, and practice variables were also added as explanatory variables in respective ways but no one variable were fit for final model.

### Statistical analysis

Univariate analysis (Chi-square test) was applied to identify the significant difference between outcome variable (KAP) and other explanatory variables. We used multinomial logistic regression to analyze factors that were associated with KAP with explanatory variables. We checked the multicollinearity among the explanatory variables using variance inflation factor (VIF). VIF value ≤ 2.0 indicates absence of multicollinearity. We performed stepwise backward likelihood ratio method and 0.05 of P-value was used as cut-point for likelihood ratio method. P-value of less than or equal 0.05 was considered as significant in both univariate and multivariate analyses. The data was summarized with relative risk ratios (RRR) and 95 % confidence interval. Completeness and accuracy of the data were checked before and after the data entry. The analysis was done in Statistical Package for the Social Sciences (SPSS) 20.0 version and R software.

### Ethical considerations

Written informed consent was taken from each participant before they enrolled for interviews. No any incentives or financial payments have been provided for the interviewed patients. Personal recognition was removed from the filled questionnaires and was assured for their confidentiality and dignity. Permission was obtained from the study setting and the study was approved by institutional ethical review committee of Nobel College, Sinamangal, Kathmandu affiliated to Pokhara University, Nepal.

## Results

Table 1 shows the sex-wise demographic characteristics of the respondents. Majority (56.6 %) of the respondents represented the age group 40–60 years and 31.1 % were above 60 years. One fifth (18 %) of the respondents were illiterate. One fourth (24.6 %) of the respondents were from rural areas and one third (32 %) of the respondents were housewife. Majority (90.6 %) of the respondents followed Hindu religion and 37.7 % were from Brahman ethnicity.

**Table 1:** Demographic characteristics by sex (n = 244)

Characteristics	Male (n = 116) n (%)	Female (n = 128) n (%)	Total (n = 244) n (%)	p- value
Age (years)				0.697
Mean ± (SD): 54.64 ± (11.76)				
≤40	13 (11.2)	17 (13.3)	30 (12.3)	
>40 - 60	64 (55.2)	74 (57.8)	138 (56.6)	
>60	39 (33.6)	37 (28.9)	76 (31.1)	
Ethnicity				0.211
Brahmin	40 (34.5)	52 (40.6)	92 (37.7)	
Kshatriya	42 (36.2)	33 (25.8)	75 (30.7)	
Others^a^	34 (29.3)	43 (33.6)	77 (31.6)	
Religion				0.030
Hindu	110 (94.8)	111 (86.7)	221 (90.6)	
Other than Hindu	6 (5.2)	17 (13.3)	23 (9.4)	
Education				<0.001
Illiterate	3 (2.6)	41 (32.0)	44 (18.0)	
Literate and up to lower secondary level	14 (12.1)	24 (18.8)	38 (15.6)	
Higher secondary level	50 (43.1)	37 (28.9)	87 (35.7)	
Graduate and above	49 (42.2)	26 (20.3)	75 (30.7)	
Residence				0.301
Rural	32 (27.6)	28 (21.9)	60 (24.6)	
Urban	84 (72.4)	100 (78.1)	184 (75.4)	
Main occupation				<0.001
Agriculture	11 (9.5)	15 (11.7)	26 (10.7)	
Business	27 (23.3)	8 (6.2)	35 (14.3)	
Service (government & private)	48 (41.4)	22 (17.2)	70 (28.7)	
Retired	30 (25.9)	5 (3.9)	35 (14.3)	
Housewife	0 (0)	78 (60.9)	78 (32.0)	

^a^Others than Brahmin & Kshatriya = indigenous caste in Nepal [38]

**Fig. 1:**
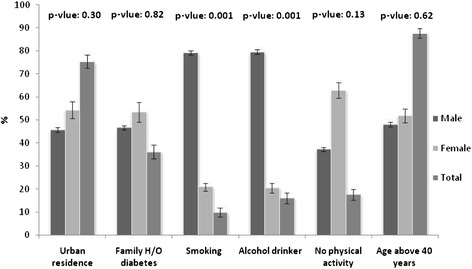
Sex-wise distribution of risk factor of diabetes

Figure 1 shows the sex-wise distribution of risk factor status of diabetes in study population. Nearly one fifth (17.6 %; among male 37.2 % vs. female 62.8 %) of the respondents had reported low or no physical activity. Overall prevalence of smoking and alcohol was 9.8 % (male 79.2 % vs. female 20.8 %, p = <0.001) and 16 % (male 79.5 vs. female 20.5 %, p = <0.001) respectively. Family history of diabetes was 36.1 % (male 46.6 vs. female 53.4 %). Majority (87.7 %) of the respondents were above 40 years (male: 48.1 % & female: 51.9 %) of age.

The different level of knowledge was as follows: 21.3 % had highly insufficient, 22.5 % had insufficient, 23 % had sufficient, 20.9 % had satisfactory and 12.3 % had highly satisfactory knowledge. Likewise, level of attitude was as follows: 28.3 % had highly insufficient, 15.2 % had insufficient, 21.3 % had sufficient, 22.5 % had satisfactory and 12.8 % had highly satisfactory attitude. Similarly, level of practice scores was as follows: 29.1 % highly insufficient, 14.8 % had insufficient, 27.9 % had sufficient, 12.3 % had satisfactory and 16.0 % had highly satisfactory practice (data not shown in Table 2).

**Table 2:** Percentage of diabetes related level of knowledge, attitude and practice

Knowledge (n = 244)	Attitude (n = 244)				
	HI (n = 69, 95 % CI)	I (n = 37, 95 % CI)	Suff (n = 52, 95 % CI)	Sat (n = 55, 95 % CI)	HS (n = 31, 95 % CI)
HI (n = 52)	28.8 (17.1,43.1)	17.3 (8.2,30.3)	15.4 (6.8,28.1)	21.2 (11.1,34.7)	17.3 (8.2,30.3)
I (n = 55)	25.5 (14.6,39.0)	20.0 (10.4,32.9)	21.8 (11.8,35.0)	20.0 (10.4,32.9)	12.7 (5.2,24.4)
Suff (n = 56)	32.1 (20.2,45.9)	12.5 (5.1,24.1)	19.6 (10.2,32.4)	23.2 (12.9,36.4)	12.5 (5.1,24.0)
Sat (n = 51)	29.4 (17.4,43.8)	7.8 (2.1,18.8)	21.6 (11.2,35.3)	25.5 (14.3,39.6)	15.7 (7.0,28.5)
HS (n = 30)	23.3 (9.9,42.2)	20.0 (7.7,38.5)	33.3 (17.2,52.8)	23.3 (9.9,42.2)	0.0
Knowledge (n = 244)	Practice (n = 244)				
	HI (n = 71, 95 % CI)	I (n = 36, 95 % CI)	Suff (n = 68, 95 % CI)	Sat (n = 30, 95 % CI)	HS (n = 39, 95 % CI)
HI (n = 52)	53.8 (39.4,67.7)	13.5 (5.5,25.7)	15.4 (6.8,28.1)	9.6 (3.2,21.0)	7.7(2.1,18.5)
I (n = 55)	27.3 (16.1,40.9)	16.4 (7.7,28.8)	32.7 (20.6,46.7)	14.5 (6.4,26.6)	9.1(3.0,19.9)
Suff (n = 56)	26.8 (15.8,40.3)	16.1 (7.6,28.3)	30.4 (18.7,44.1)	8.9 (2.9,19.6)	17.9(8.9,30.4)
Sat (n = 51)	19.6 (9.8,33.1)	13.7 (5.7,26.2)	29.4 (17.4,43.8)	11.8 (4.4,23.8)	25.5(14.3,39.6)
HS (n = 30)	10.0 (2.1,26.5)	14.8 (3.7,30.7)	33.3 (17.2,52.8)	20.0 (7.7,38.5)	23.3(9.9,42.2)
Attitude (n = 244)	Practice (n = 244)				
	HI (n = 71, 95 % CI)	I (n = 36, 95 % CI)	Suff (n = 68, 95 % CI)	Sat (n = 30, 95 % CI)	HS (n = 39, 95 % CI)
HI (n = 69)	26.1 (16.2,38.1)	13.0 (6.1,23.3)	31.9 (21.1,44.2)	10.1 (4.1,19.8)	18.8 (10.4,30.1)
I (n = 37)	43.2 (27.1,60.5)	18.9 (7.9,35.1)	16.2 (6.1,32.0)	13.5 (4.5,28.7)	8.1 (1.7,21.9)
Suff (n = 52)	15.4 (6.8,28.1)	13.5 (5.5,25.7)	34.6 (21.9,49.1)	15.4 (6.8,28.1)	21.2 (11.1,34.7)
Sat (n = 55)	30.9 (19.1,44.8)	16.4 (7.7,28.8)	21.8 (11.8,35.0)	10.9 (4.1,22.2)	20.8 (10.4,32.9)
HS (n = 31)	38.7 (21.8,57.8)	12.9 (3.6,29.8)	32.3 (16.6,51.3)	12.9 (3.6,29.8)	3.2 (0.1,16.7)

The total median KAP scores were 81 (59–89), 40 (32–40) and 14 (4–20) respectively. For knowledge 78, 80, 83, 85 attitude 38, 39, 40, 42 and practice 12, 13, 15, 16 were the quintile (first to fourth) scores. Then quintile scores were obtained. The level of KAP was classified into the following five categories based on the quintile score coded as: ≤20 % highly insufficient, 21-40 % insufficient, 41-60 % sufficient, 61-80 % satisfactory and >80 % highly satisfactory [27, 28]

*CI* confidence interval, *HI* highly insufficient, *I* insufficient, *Suff* sufficient, *Sat* satisfactory, *HS* highly satisfactory

Those respondents who had highly sufficient knowledge, none had highly satisfactory attitude whereas 23.3 % had highly satisfactory practice. Those respondents who had highly insufficient knowledge, 17.3 % had highly satisfactory attitude and 7.7 % had highly satisfactory practice. Similarly, among those who had sufficient knowledge, 19.6 % had sufficient attitude, 23.2 % had satisfactory attitude, 12.5 % had highly satisfactory attitude and 8.9 % and 17.9 % had satisfactory practice and highly satisfactory practice respectively (Table 2).

The highly satisfactory attitude decreased from 17.3 % to 0 % with the increased level of knowledge. Likewise, when the level of knowledge increased, the highly satisfactory practice increased from 7.7 % to 25.5 % and again decreased to 23.3 % among those who have highly satisfactory knowledge. Among those who have highly insufficient attitude, 18.8 % had highly satisfactory practice. When the attitude was sufficient, 15.4 % had highly insufficient practice and 21.2 % had highly satisfactory practice. There was variation on highly satisfactory practice with the increased level of attitude (Table 2).

Respondents of age group 41 to 60 years had highly satisfactory knowledge (66.7 % vs 59.0 %) and practice than those in other age group. Female were more likely to have highly satisfactory knowledge (53.3 % vs 59.0 %) and practice than males. Brahmins had highly satisfactory knowledge (40 %) and attitude (46.1 %) than that of Kshatriya being (20 %). Hindu had highly satisfactory knowledge (83.3 %) and practices (89.7 %). Respondents with higher secondary education had highly sufficient knowledge (43.3 %) and attitude (30.8 %) and respondents involved in service (government and private) had highly sufficient knowledge (40 %) but housewife (38.5 %) had highly satisfactory practice than those involved in service (20.5 %) (Data not shown in Table 2).

Statistically significant difference was found in the level of knowledge to age (p = 0.006), education (p = <0.001) and family history of diabetes (p = 0.003). Next, there was significant difference in the level of attitude and education (p = 0.012). Conversely, the level of practice was statistically different to sex (p = 0.049) and family history of diabetes (p = 0.050). There was no significant difference in the level of knowledge (p = 0.171), attitude (p = 0.209) and practice (p = 0.483) in comparison with occupation (Data not shown in Table 2).

Table 3 presents the multinomial regression analysis with demographic characteristics, and risk factors with KAP. Highly insufficient was the reference group. The association of having a satisfactory level of knowledge among the respondents those who have age > 40–60 years was 5.07 times greater than among those who were of age ≤ 40 years. Increasing levels of respondent’s education have significant increasing level of highly satisfactory knowledge vs. highly insufficient level of knowledge. The relative probability of having a highly satisfactory level of knowledge was 7.53 times higher among those who did physical activities than those who did not do physical activities.

**Table 3:** Multinomial logistic regression with background variable and Knowledge (n = 244)

Characteristics	HI vs. I	HI vs Suff	HI vs. Sat	HI vs. HS
	RRR (95 % CI)	RRR (95 % CI)	RRR (95 % CI)	RRR (95 % CI)
Age (years)				
≤40	-	Reference	Reference	Reference
>40 - 60	-	2.7 (0.82,8.9)	5.07 (1.36,18.91)*	2.96 (0.76,11.59)
>60	-	1.38 (0.39,4.92)	2.24 (0.54,9.34)	0.63 (0.12,3.32)
Sex				
Female	Reference	Reference	Reference	Reference
Male	0.33 (0.14,1.13)*	1.05 (0.38,2.91)	0.39 (0.14,1.09)	0.49 (0.15,1.56)
Religion				
Hindu	Reference	Reference	Reference	Reference
Other than Hindu	0.43 (0.11,1.68)	0.2 (0.04,1.12)	0.16 (0.03,0.92)*	0.96 (0.23,4.05)
Education level				
Illiterate	Reference	Reference	Reference	Reference
Literate and up to lower secondary	12.42 (2.82,54.63)**	1.6 (0.35,7.38)	14.72 (2.77,78.32)**	1.83 (0.23,14.68)
Higher secondary	23.3 (5.54, 98.06)**	5.8 (1.56,21.5)**	19.6 (3.84,100.09)**	10.57 (1.98,56.41)**
Graduate and above	22.12 (4.86,100.77)**	6.28 (1.55,25.36)**	14.43 (7.8,220.08)**	16.79 ( 2.96,95.2)**
Physical activities				
No	Reference	Reference	Reference	Reference
Yes	2.59 (0.88,7.6)*	3.78 (1.31,10.9)*	3.02 (0.99,9.24)	7.53 (1.51,37.71)*
Attitude (n = 244)				
Education level				
Illiterate	Reference	Reference	Reference	Reference
Literate and up to lower secondary	0.31 (0.08,1.16)	1.17 (0.32,4.3)	0.86 (0.23,3.16)	2.0 (0.42,9.51)
Higher secondary	0.98 (0.37, 2.63)	3.0 (0.98,9.17)	2.04 (0.68,6.09)	1.75 (0.39,7.91)
Graduate and above	0.18 (0.04,0.76)*	1.97 (0.62,6.32)	2.59 (0.88,7.66)	3.95 (0.96,16.2)
Practice (n = 244)				
Sex				
Female	Reference	Reference	Reference	Reference
Male	1.69 (0.68,4.18)	1.51 (0.71,3.21)	0.43 (0.16,1.19)	0.77 (0.3,1.96)
Smoking				
No	Reference	Reference	Reference	Reference
Yes	0.18 (0.03,0.95)*	0.3 (0.09,0.93)*	0.35 (0.07,1.85)	0.1 (0.01,0.92)*
Duration of diabetes				
0.5-5 Years	Reference	Reference	Reference	Reference
>5-10 Years	4.44 (1.22,16.15)*	2.66 (0.87,8.17)	1.5 (0.39,5.87)	7.26 (2.06,25.56)**
>10 Years	3.17 (1.16,8.65)*	2.21 (0.93,5.27)	1.41 (0.45,4.46)	4.36 (1.56,12.24)**
Physical activities				
No	Reference	Reference	Reference	Reference
Yes	2.78 (0.89,8.62)	5.27 (1.93,14.42)**	9.16 (1.95,43.1)**	3.74 (1.09,12.87)*

*RRR* relative Risk Ratio, *CI* confidence interval, *HI* highly insufficient, *I* insufficient, *Suff* sufficient, *Sat* satisfactory, *HS* highly satisfactory

* ≤ 0.05, ** ≤ 0.01

The relative probability of having an insufficient level of attitude (0.18) among respondents who have graduate and above level of education was significantly lower than those who were illiterate (Table 3).

The association of having an insufficient level of practice (0.18), a sufficient level of practice (0.3) and a highly sufficient level of practice (0.1) were significantly lower among those who were smoker than those who did not. The association of having a highly sufficient level of practice was 7.26 times higher among those who were suffered from diabetes from >5-10 years and 4.36 times higher among those who were suffered from diabetes from more than 10 years. The relative probability of having a highly satisfactory level of practice was 3.74 times higher among those who did physical activities than those who did not do physical activities (Table 3).

## Discussion

Diabetes is a chronic disease with different level of complication that requires broad self-care knowledge and management. Extensive knowledge, attitude and good practice could be the means to control and prevent diabetes related consequences and cost effective measures in LMICs. Our study reveals the poor level of overall knowledge, attitude and practice (KAP) among diabetic patients. Previous studies from Asia and middle-east revealed that the knowledge related to diabetes was poor among people with diabetes [17, 21, 22, 29, 30]. Some studies reported that diabetic patients had good level of diabetes related knowledge [31–33]. Albeit the comparison of our result with other studies are difficult because of nature of the study population and applied measurements were dissimilar. Our finding revealed there is variation in KAP among diabetic patients. For example practice is increased with increased in knowledge and attitude is decreased with increased in knowledge. Likewise, when the level of attitude is increased, the level of practice is decreased. This variation could be due to lack of motivation to apply the knowledge into action or practice and conservative thoughts with over confidence. Our finding revealed that the type of diabetes and level of KAP was not found statistically significant difference. We could not establish the comparison with existing literatures due to the unavailability of published literatures. The possible reason might be the perceived risk factors were similar among both type of diabetic patients. However the pathological and other characteristics would be different.

Further, in case of attitude, our study finding showed the poor level of attitude, while other studies from urban area of South India and UAE reported the contradictory findings that positive and good level of attitude among diabetic patients [21, 30]. Significant association in level of knowledge to age, education and family history of diabetes was reported in our study. This result is similar with the KAP study of diabetes in Bangladesh and UAE which stated that knowledge was significantly associated with level of education [21, 22]. However, in contrast, our study revealed that increased level of education was significantly associated with insufficient level of attitude. Similarly the findings from Bangladesh and UAE reported that education was not significantly associated with the level of attitude towards diabetes [21, 22]. Conventional careless thoughts with over confidence might be the possible reason that they did not change their attitude toward diabetes care among educated people.

The likelihood of good practice among female was more as compared to male. This findings remain contradict with the previous studies from Africa, Asia and Middle East [32, 34–36]. The increased duration of diabetes was associated with good practice in our study which was similar to Nigerian and Ethiopian findings [35, 37].

Smoking is accountable for NCDs related death [15]. Our study reported 9.8 % of smoker’s among those who were diabetic. Further in our study, smoker had reported low or no physical activities which may further increase the consequences of diabetes. Similar finding was reported for Arabian people [21]. Furthermore, previous finding suggested that 27 % of the diabetes are caused by physical inactivity [15]. Approximately, 20 min daily moderate physical activity can reduce 27 % risk of diabetes [15, 16]. Despite the fact that physical inactivity is the leading risk factor among the NCDs and diabetes. However, nearly one fifth of those with diabetes reported low or no physical activity in this study. Simultaneously alcohol consumption is also another associated risk factor with diabetes [15]. Nearly 16 % of respondents in our study had habits of alcohol consumption which may increases severity of diabetics.

Our study revealed that most of the patients have been diagnosed before the age of 40 years. Similarly, previous report showed that the higher number of diabetes was seen among the age of 40–49 years [2]. Burden of diabetes is increasing in trend and need to start screening with the early age of life. Furthermore, the possible reason for increasing burden could be the increase in risk factors including physical inactivity, smoking, harmful use of alcohol, changing in food habit and modification in lifestyles gradually with modernization and urbanization. Therefore our study notifies the necessity to improve education strategies and develop modern tools that improve diabetes related consequences and hazards. For this improvement and efforts, further intervention would require to focus on health related knowledge, attitude and practices among diabetic patients.

### Strength and limitations

We had worth noting limitations in this study. Our study setting was institutional based therefore the finding may not be generalize for other diabetic population. The study design and sampling technique we used could not establish valid causality of the association among other variables. Socio economic status or income is the social determinant of disease outcome, but we could not use it in our study because the respondent did not provide complete information. We couldn’t measure all the risk factor especially BMI because our major objective was to assess the KAP of the respondents. We measured variation in KAP among early and late diagnosed patient. In fact, public health related problems need to observe and require different settings and approaches among diabetes affected group.

## Conclusion

Our institutional based cross-sectional study revealed diabetes related poor health knowledge, improper attitude and poor practice among those who are affected by diabetes in low income country Nepal. This study highlighted the factors that we need to consider while developing health promotion activities. Further, health literacy, counseling and education program need to be develop in both clinical and community settings. Our results show the potential diabetes health literacy needs to be improved or developed for improved health promotion.
